# Attentional salience and the neural substrates of response inhibition in borderline personality disorder

**DOI:** 10.1017/S0033291721000118

**Published:** 2022-11

**Authors:** J. S. Wrege, D. Carcone, A. C. H. Lee, C. Cane, U. E. Lang, S. Borgwardt, M. Walter, A. C. Ruocco

**Affiliations:** 1 Department of Psychiatry, University Psychiatric Clinics of Basel, Wilhelm Klein-Strasse 27, CH-4002 Basel, Switzerland; 2 Department of Psychology, University of Toronto, Toronto, Ontario, Canada; 3 University of Basel, Basel, Switzerland

**Keywords:** Borderline personality disorder, functional magnetic resonance imaging, impulsivity, no-go, response inhibition, saliency

## Abstract

**Background:**

Impulsivity is a central symptom of borderline personality disorder (BPD) and its neural basis may be instantiated in a frontoparietal network involved in response inhibition. However, research has yet to determine whether neural activation differences in BPD associated with response inhibition are attributed to attentional saliency, which is subserved by a partially overlapping network of brain regions.

**Methods:**

Patients with BPD (*n* = 45) and 29 healthy controls (HCs; *n* = 29) underwent functional magnetic resonance imaging while completing a novel go/no-go task with infrequent odd-ball trials to control for attentional saliency. Contrasts reflecting a combination of response inhibition and attentional saliency (no-go > go), saliency processing alone (oddball > go), and response inhibition controlling for attentional saliency (no-go > oddball) were compared between BPD and HC.

**Results:**

Compared to HC, BPD showed less activation in the combined no-go > go contrast in the right posterior inferior and middle-frontal gyri, and less activation for oddball > go in left-hemispheric inferior frontal junction, frontal pole, superior parietal lobe, and supramarginal gyri. Crucially, BPD and HC showed no activation differences for the no-go > oddball contrast. In BPD, higher vlPFC activation for no-go > go was correlated with greater self-rated BPD symptoms, whereas lower vlPFC activation for oddball > go was associated with greater self-rated attentional impulsivity.

**Conclusions:**

Patients with BPD show frontoparietal disruptions related to the combination of response inhibition and attentional saliency or saliency alone, but no specific response inhibition neural activation difference when attentional saliency is controlled. The findings suggest a neural dysfunction in BPD underlying attention to salient or infrequent stimuli, which is supported by a negative correlation with self-rated impulsiveness.

## Introduction

Borderline personality disorder (BPD) is a psychiatric disorder characterized by impulsive behaviors, such as substance abuse and self-harm (American Psychiatric Association, 2013). Impulsivity is a core symptom of BPD that is stable across time and predicts BPD psychopathology over several years (Links, Heslegrave, & Reekum, 1999). The cognitive processes underlying impulsivity in BPD have not yet been fully identified, although deficits in so-called ‘executive functions’ that facilitate goal-directed behaviors are likely candidates (Koudys, Traynor, Rodrigo, Carcone, & Ruocco, 2019). Deficits in multiple related executive functions have been implicated in BPD, including in response inhibition, cognitive flexibility, decision-making, problem-solving, and planning (Paret, Jennen-Steinmetz, & Schmahl, 2017; Ruocco, 2005; Unoka & Richman, 2016). The most commonly studied executive function in neuroimaging studies of BPD is response inhibition, referring to ‘the ability to suppress inappropriate, irrelevant, or suboptimal actions’ (Verbruggen, 2017, p. 1). However, only a small number of these studies has investigated response inhibition independent of any emotional context, that is, without incorporating emotionally-valenced words or images. The go/no-go (GNG) paradigm has been used most frequently in neuroimaging studies of BPD, as the task has classically been used to investigate the neural structures and functions involved in response inhibition in healthy and clinical populations (Aron, Robbins, & Poldrack, 2014; Drewe, 1975).

Until now, neuroimaging research on response inhibition in BPD has typically applied variants of a conventional GNG paradigm, which contrasts infrequent no-go stimuli with frequent go stimuli. Despite greater self-reported impulsiveness, patients with BPD show no discernible neural activation differences compared to healthy controls (HCs) associated with no-go response inhibition (Mortensen, Rasmussen, & Håberg, 2010; Silbersweig et al., 2007; van Eijk et al., 2015). The exception is an event-related potential study using a hybrid flanker-GNG paradigm, which found a lower no-go P3 amplitude in BPD compared to HC (Ruchsow et al., 2008). Neuroimaging studies employing the typical GNG paradigm, however, conflate the neural signal associated with response inhibition and the activation related to the salience of the infrequent no-go stimuli. According to Corbetta and Shulman (2002), the latter process is supported by a right-lateralized network specialized for the detection of behaviorally relevant stimuli, which includes events that are salient or occur infrequently. This ventral frontoparietal network, which includes the inferior frontal gyrus (IFG), middle frontal gyrus, and frontal operculum, functions to reorient attention. Response (or motor) inhibition is also linked to the right ventrolateral prefrontal cortex (vlPFC); (Levy & Wagner, 2011), although different subregions subserve distinct functions: the inferior frontal junction (IFJ) – lying between the inferior frontal sulcus and precentral sulcus – detects infrequent stimuli, whereas the posterior IFG (pIFG) mediates response inhibition (Chikazoe, 2010) together with the supplementary motor area (SMA), pre-SMA, and subthalamic nucleus (Chambers, Garavan, & Bellgrove, 2009). As the right vlPFC is sensitive to both salience (or infrequency) and response inhibition processes (Walther, Friederich, Stippich, Weisbrod, & Kaiser, 2011), it is imperative to differentiate their neural underpinnings and comparative dysfunctions in BPD.

In the present study, we sought to address the aforementioned limitations of the typical GNG paradigm to elucidate dissociable patterns of neural activation associated with attentional saliency and response inhibition in patients with BPD and age- and sex-matched HC. We applied a modified GNG paradigm to control for attentional saliency by implementing a third trial type: infrequent visual oddballs (Rubia et al., 2006; Rubia, Smith, Brammer, & Taylor, 2003). The oddball trials are presented as infrequently as no-go trials (but requiring a response), allowing us to more directly isolate brain activity underlying attentional saliency and response inhibition. Differentiating the neural substrates of these partially overlapping processes is crucial for clarifying the nature of the neural dysfunctions underlying impulsive behaviors in BPD, especially given that prior functional magnetic resonance imaging (fMRI) research using the typical GNG paradigm has detected no neural activation differences compared to healthy individuals (Mortensen et al., 2010; van Eijk et al., 2015). Studies employing an infrequent oddball comparison show that the no-go > oddball contrast is associated with brain activation in the mesial frontal and right IFG, anterior cingulate cortex (ACC), right caudate, left temporal cortex, precuneus, bilateral insula, and right sensorimotor cortex (Rubia et al., 2006; Schmitz et al., 2006).

While prior research has not revealed neural activation differences between BPD and HC on typical GNG tasks (Mortensen et al., 2010; van Eijk et al., 2015), it is possible that the studies were underpowered to detect potential group differences (BPD and HC groups each ranged from *n* = 15–18). Furthermore, the studies did not consider the impact of no-go trial infrequency on neural activation (e.g. using an oddball trial requiring a response), obfuscating potentially distinctive activation patterns associated with attentional saliency *v.* response inhibition. Given that patients with BPD report heightened impulsivity and inattention (Davids & Gastpar, 2005; Links et al., 1999; Ruocco et al., 2019), we anticipated differences in neural activation between BPD and HC in brain regions related to response inhibition (no-go > go) and attentional saliency (oddball > go). Specifically, we expected lower engagement in BPD of response inhibition-related (no-go > go) brain areas, namely, the rIFG, SMA, pre-SMA (Chambers et al., 2009; Chikazoe, 2010), whereas for saliency (oddball > go), we expected lower activation in the frontal pole, middle frontal gyrus, frontal operculum, medial and mesial brain areas, and ACC (Corbetta & Shulman, 2002; Criaud & Boulinguez, 2013; Mostofsky & Simmonds, 2008; Simmonds, Pekar, & Mostofsky, 2008). However, we did not have *a priori* hypotheses regarding the direct comparison of response inhibition and attentional saliency (no-go > oddball), as research has yet to consider these processes jointly in BPD. Finally, we anticipated that neural activation associated with response inhibition and attentional saliency would be associated with self-rated impulsiveness and inattention, respectively.

## Method

### Eligibility criteria

Participants with BPD were required to meet Diagnostic and Statistical Manual of Mental Disorders – Fourth Edition (DSM-IV) (American Psychiatric Association, 2013) criteria based on the Structured Clinical Interview for DSM-IV-TR – Axis II Disorders (SCID-II) – German version (Wittchen, Wunderlich, Gruschwitz, & Zaudig, 1997). They were deemed ineligible to participate if they reported acute psychotic symptoms. Exclusion criteria for HC were a current or prior history of a DSM-IV Axis I or Axis II disorder. All participants were at least 18 years of age and capable to give informed consent for participation in the study. Alcohol and other substance use (except nicotine) was not allowed within 1 week before the scan day.

### Participant characteristics

Participants with BPD (*n* = 45) were consecutively recruited from an inpatient treatment program in the Psychiatric University Hospital (UPK Basel) in Switzerland. The SCID-II was administered by either a licensed psychiatrist or clinical psychology trainee supervised by a licensed psychiatrist. Information from the Axis I, including the attention-deficit hyperactivity disorder (ADHD) module, and Axis II diagnostic interviews was reviewed in a diagnostic meeting to arrive at consensus diagnoses for BPD and other psychopathologies. The HC participants (*n* = 29) were recruited using internet advertisements and after being matched to BPD participants for age and sex, underwent the same diagnostic procedures. Table 1 depicts participants' sociodemographic characteristics.

**Table 1.** Sociodemographic and clinical data

	BPD *N* = 45 (mean, s.d., *n*)	HC *N* = 29 (mean, s.d., *n*)	Significance
Sex			Fisher's exact test, *p* = 0.55
Female	35 (77.8%)	25 (86.2%)	
Male	10 (22.2%)	4 (13.8%)	
Age	27.51 (8.03)	25.69 (6.04)	*t* = −0.66, *p* = 0.51
IQ	97.55, 6.48	104.38, 12.04	*t* = −2.77, *p* < 0.01
Axis I disorders			
Depressive disorders	18 (40%)	0 (0%)	**–**
Anxiety disorders	7 (15.6%)	0 (0%)	**–**
Eating disorders	7 (15.6%)	0 (0%)	**–**
Somatoform disorders	2 (4.4%)	0 (0%)	**–**
Posttraumatic stress disorder	3 (6.7%)	0 (0%)	**–**
Substance disorders	17 (37.8%)	0 (0%)	**–**
Medication	26 (57.8%)	0 (0%)	**–**
Global assessment scale – GAS	43.16 (8.56)	–	**–**
Beck depression inventory – BDI	26.32 (12.15)	2.38 (2.49)	*t* = −7.16, *p* < 0.01
Borderline symptom list 23 – BSL-23	1.78 (0.90)	0.19 (0.19)	*t* = −6.83, *p* < 0.01
Barratt Impulsiveness			
Scale – BIS-11: sum	68.93 (11.68)	56.71 (9.51)	*t* = 4.89, *p* < 0.01
Attention	19.73 (3.53)	14.18 (3.01)	*t* = 7.18, *p* < 0.01
Motor	22.73 (4.76)	20.79 (3.01)	*t* = 2.14, *p* < 0.04
Non-planning	26.47 (5.48)	21.75 (5.02)	*t* = 3.77, *p* < 0.01

*Note.* Beck depression inventory: A cut-off score of 18 indicates clinical relevance (Hautzinger, Bailer, Worall, & Keller, 1995). Borderline symptom list 23: Validation sample of BPD patients showed *M* = 2.05, s.d. = 0.90 (Wolf et al., 2009). IQ measured with multiple choice vocabulary test – German version [MWT-B; Lehrl, Triebig, and Fischer (2005)].

Of the 45 participants with BPD, 26 were under stable drug regime before scanning. Medications included antidepressants (Trimipramine, *n* = 2; Mirtazapine, *n* = 1, Trazodone, *n* = 2; Fluoxetine, *n* = 3; Escitalopram, *n* = 4; Duloxetine, *n* = 5; Venlafaxine, *n* = 1; and Agomelatine, *n* = 3), mood stabilizer (Lamotrigine, *n* = 2), antipsychotics (Chlorprothixene, *n* = 3; Quetiapine, *n* = 1; and Aripiprazole, *n* = 1), and stimulants (Methylphenidate, *n* = 1).

### Procedures

During the first week of inpatient treatment, patients were approached to determine their interest in participating in the study. All participants provided written informed consent and received a copy of the study description. Until the end of the week, all participants completed magnetic resonance imaging (MRI) safety screening protocols and psychometric assessment (questionnaires and interviews). On the scan day, directly prior to the MRI scan, participants completed a urine toxicology screen and a breathalyzer test to exclude individuals with possible substance intoxication. The scanning protocol included tasks other than the GNG task (Wrege et al., 2019), the results of which are not reported in the present article.

### Self-report measures

Overall, the psychometric assessment in both groups included multiple symptom measures: Borderline Symptom List [BSL-23; Bohus et al. (2009)]; German Version of the Beck Depression Inventory [BDI; Beck, Ward, Mendelson, Mock, and Erbaugh (1961)]; German version of the Barratt-Impulsiveness-Scale [BIS-11; Preuss et al. (2003)]. The second-order subfactors of the BIS-11 were calculated according to Patton, Stanford, and Barratt (1995), which include attentional impulsivity, motor impulsivity, and non-planning impulsivity.

### Magnetic resonance imaging

#### fMRI go/no-go paradigm

**Fig. 1.**
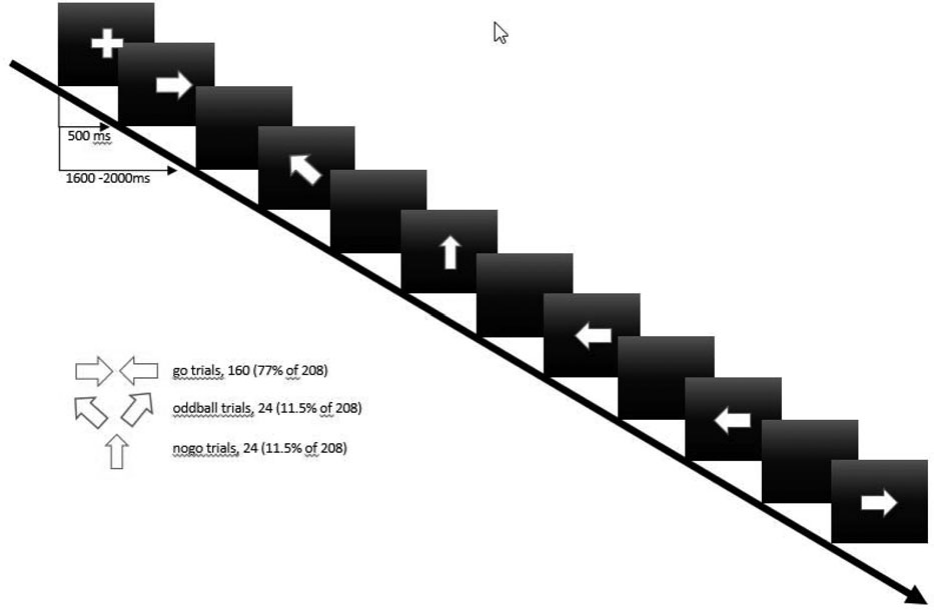
Screen set-up for the scanner.

The event-related GNG paradigm incorporated three types of stimuli: 160 (77%) go trials, 24 (11.5%) no-go trials, and 24 (11.5%) oddball trials. The inter-stimulus interval (ISI) was jittered between 1600 and 2000 ms (mean ISI 1800 ms), and the total duration of the paradigm was ~6 min. The task was self-paced and all stimuli were presented for a maximum of 500 ms. The task requires the execution (on go and oddball trials) or inhibition (on no-go trials) of a motor response (button press) with the right hand depending on what stimulus was visually presented (Rubia et al., 2006). Participants were instructed to respond as quickly and accurately as possible. During go trials, an arrow pointing to the left or right was presented and participants had to press the left or right button accordingly. On no-go trials an upward pointing arrow was presented and participants were instructed to withhold their response. To control for novelty effects associated with the low frequency and orientation of no-go compared to go trials, oddball trials were implemented and consisted of an arrow pointing to the left or right at a 23° angle. Participants were instructed to respond in the same way to the infrequent oddball stimuli as for go trials. For all trial types, the arrow was 3 cm in size and white in color and presented in the center of the screen against a black background (Fig. 1).

#### fMRI image acquisition and analysis

Participants were scanned using a 3T MRI system (Siemens Magnetom Prisma, Erlangen, Germany) and a 20-channel phased-array radio frequency head coil. Scanning was conducted with the following parameters: interleaved acquisition, 39 axial slices of 3 mm thickness, 0.5 mm interslice gap, the field of view 228 × 228 mm^2^ and an in-plane resolution of 3 × 3 mm^2^. Repetition time was 2.5 s, echo time was 30 ms, and the bandwidth was set to 2350 Hz/pixel. The total run time was 6 min, yielding a maximum total of 160 volumes (depending on the individual's speed at completing the task). To ensure signal stabilization, we acquired two dummy scan volumes. Stimulus presentation and recording of responses as well as reaction times were carried out with E-Prime^®^ using an MRI-compatible response box.

Analysis of fMRI data was performed with FMRI Expert Analysis Tool (FEAT) version 6.00 and other software included in the FMRIB Software Library (FSL Version 5.0.8; http:/www.fmrib.ox.ac.uk/fsl; Jenkinson, Beckmann, Behrens, Woolrich, and Smith, 2012). The first two dummy scans were deleted. Motion correction was performed with respect to the first volume using linear (affine) registration as implemented by the MCFLIRT tool (Jenkinson, Bannister, Brady, & Smith, 2002). Non-linear normalization into standard stereotactic space with 12 degrees of freedom ensued with respect to the Montreal Neurological Institute 152 – (MNI152) template. For spatial smoothing, we applied a Gaussian kernel of 6 mm full-width half-maximum. The onset times of go, no-go, and oddball trials were modelled for a duration of 500 ms convolved with a double gamma function, and time derivatives were included into the model. On the first-level, subject-specific condition effects as well as between condition contrasts were defined as follows: go, oddball, no-go, response inhibition *v.* active baseline (no-go > go), attentional saliency (oddball > go), motor response inhibition controlling for effects of saliency/infrequency (no-go > oddball). We computed parameter estimates for propagation into second-level group analyses (BPD > HC, HC > BPD, BPD mean, HC mean) of mixed effects using FLAME 1 – FMRIB's Local Analysis of Mixed Effects (Beckmann, Jenkinson, & Smith, 2003). Given statistical concerns of inflated false-positive rates for the use of cluster-wise inference (Beckmann et al., 2003), non-parametric permutation testing (*n* = 5000) employing threshold-free cluster enhancement [TFCE, Smith and Nichols (2009)] via the FSL ‘randomize’ function was applied (Winkler, Ridgway, Webster, Smith, & Nichols, 2014). Correction was set to *α* = 0.05 for FWE. Significant activation was subsequently identified using the Oxford-Harvard Cortical and Subcortical Atlases.

To examine the relationship between self-ratings and significant brain activity, we extracted individual mean percent-signal change using Featquery in FSL (Jenkinson et al., 2012) by centering a 5 mm sphere on the maximum voxel of the significant clusters of interest. Extracted data were then correlated with self-ratings using Statistical Package for Social Sciences (SPSS 24).

## Results

### Participant characteristics and behavioral performance

Table 1 displays psychiatric diagnostic comorbidity and symptom information for the participant samples as well as comparisons between the groups.

Performance was operationalized as percent correct responses over all trials (go, oddball, no-go), and did not significantly differ between the groups (*F* = 2.904, *p* = 0.093, df = 72), although there was a trend toward BPD patients having poorer performances (BPD_mean_ = 0.92% correct, s.d. = 0.079; HC_mean_ = 0.95% correct, s.d. = 0.059). The descriptive statistics of correct responses for each trial type were as follows: go trials: BPD = 92.4% (s.d. = 0.08), HC = 94.9% (s.d. = 0.06); oddball trials: BPD = 87.2% (s.d. = 0.14), HC = 89.4% (s.d. = 0.14); no-go trials: BPD = 94.7% (s.d. = 0.07), HC = 97.4% (s.d. = 0.03). We calculated a two-way-ANOVA with the group as factor one and trial types as factor two. The overall multivariate model was not significant (Pillai-Spur = 0.06 *F* = 1.484 df_1_ = 3 df_2_ = 70 *p* = 0.226). Similarly, there was no significant between-groups effect (go: corrected *R*^2^ = 0.014 *F* = 2.034 *p* = 0.158; oddball: corrected *R*^2^ = −0.007 *F* = 0.468 *p* = .496; no-go: corrected *R*^2^ = 0.031 *F* = 3.353 *p* = 0.071).

Symptom load, as measured with BSL-23, correlated (Pearson's *r*) negatively with the task performance (BPD *r* = −0.322 *p* = 0.03, HC *r* = −0.427 *p* = 0.02, groups collapsed *r* = −0.342 *p* < 0.01). Self-rated attentional impulsiveness measured with the BIS-11 attention subscale also correlated (Pearson's *r*) negatively with the performance in the GNG task when both groups were collapsed (groups collapsed *r* = 0.−301 *p* = 0.01, BPD *r* = −0.27 *p* = 0.07, HC *r* = −0.17 *p* = 0.38).

### Main imaging results: whole brain results of second-level between-group effects

**Fig. 2.**
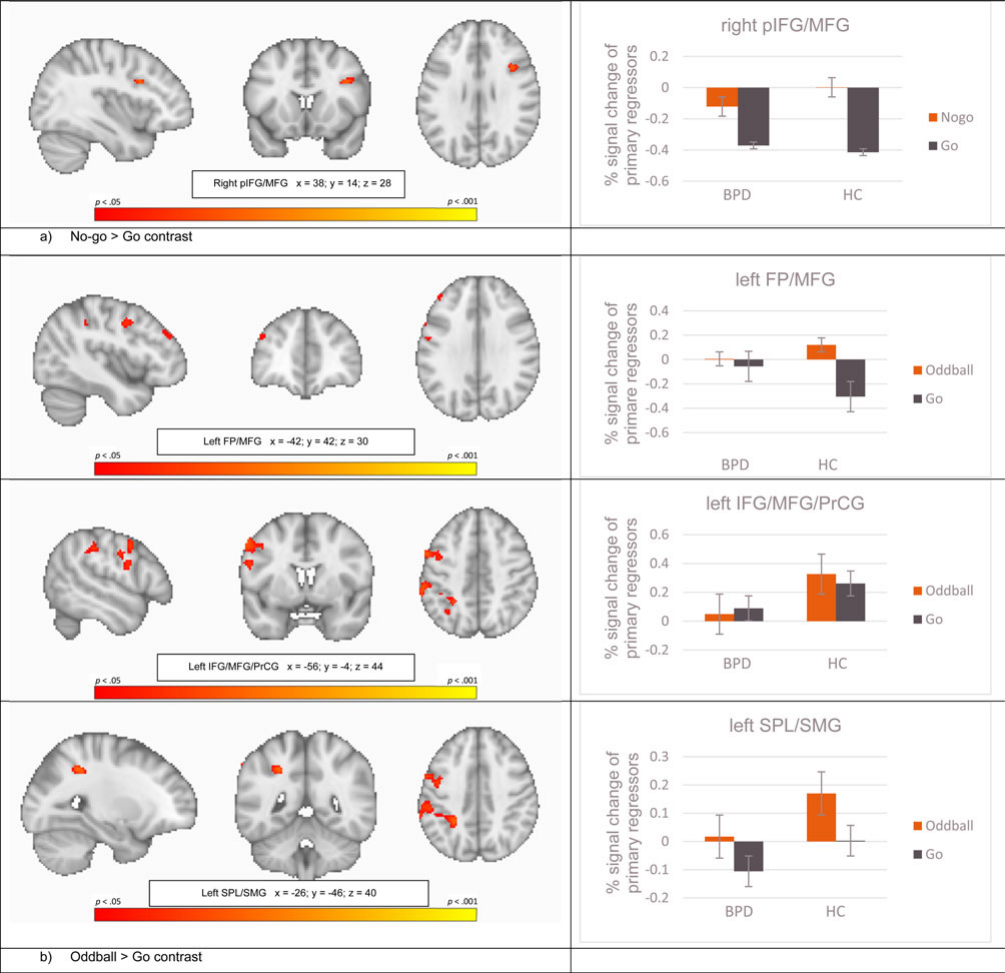
Column 1 Randomized t-contrast maps, and column 2 percent-signal changes of the primary regressors (lines depict standard deviations). *Note*. FP, frontal pole; pIFG, posterior inferior frontal gyrus; MFG, middle frontal gyrus; PrCG, precentral gyrus; SMG, supramarginal gyrus; SPL, superior parietal lobule. (*a*) No-go > go contrast, (*b*) Oddball > go contrast.

Non-parametric statistics revealed significant brain activation differences between HC and BPD in the combined saliency and response inhibition contrast (no-go > go) and in the saliency alone contrast (oddball > go), reflecting greater activity in HC compared to BPD in all significant clusters. During the combined contrast of response inhibition and saliency (no-go > go), this included a cluster comprising the right inferior and middle frontal gyri and the right precentral gyrus (rIFG/MFG/PrCG; voxels = 58; *t* = 5.17; *p* = 0.03; peak: *x*= 38, *y* = 14, *z* = 28) (Fig. 2a). In the saliency alone contrast (oddball > go), HC showed significant greater activity in three clusters: left frontal pole and left middle frontal gyrus (lFP/MFG; voxels = 46; *t* = 4.10; *p* = 0.04; peak: *x* = −42, *y* = 42, *z* = 30), left inferior and middle frontal and precentral gyri (lIFG/MFG/PrCG; voxels = 411; *t* = 4.34; *p* = 0.03; peak: *x* = −56, *y* = 4, *z* = 44), and left superior parietal lobe and supramarginal gyrus, postcentral gyrus, and lateral occipital and angular gyri (lSPL/SMG/PoCG/LOG/AG; voxels = 467; *t* = 4.47; *p* = 0.03; peak: *x* = −26, *y* = −46, *z* = 40) (Fig. 2b). Full details of these significant clusters can be found in Table 2a. There was no significant brain activation for the opposite group-level contrast (BPD > HC). The saliency-controlled response inhibition contrast (no-go > oddball) did not reveal any significant differences in activation between-groups.

**Table 2.** Significant non-parametric between-group brain activations

						*k*_E_			
HC > BPD^a^		Hemisphere				cluster	MNI (*x*)	MNI (*y*)	MNI (*z*)
(a) First-level contrasts (no-go > go, no-go > oddball, oddball > go)									
No-go > go		R	IFG	MFG	PrCG	58	38	12	28
No-go > odd		–	–	–	–	–	–	–	–
Odd > go		L	FP	MFG		46	−42	42	30
		L	IFG	MFG	PrCG	411	−56	4	44
		L	SPL/SMG	PoCG	lOG/AG	467	−26	−46	40
(b) First-level regressors (go, oddball, no-go)									
Go		–	–	–	–	–	–	–	–
Odd		L	IFGpo	MFG	PrCG	68	−40	8	30
		L	SMG/PoCG	SPL/AG	Precuneus/LOG	2696	−30	−40	36
No-go		L	SPL	Precuneus	PoCG	25	−16	−52	54
		L	SMG	PoCG	SPL	119	−28	−40	36
		L	PrCG	PoCG		141	−52	−6	34

AG, angular gyrus; FP, frontal pole; IFGpo, inferior frontal gyrus, pars opercularis; LOG, lateral occipital gyrus; MFG, middle frontal gyrus; Pr/PoCG, precentral/postcentral gyrus; SMG, supramarginal gyrus; SPL, superior parietal lobe.

*Note:* Co-ordinates in MNI space (*x y z*) of peak voxel and cluster size (*k*_E_ cluster). Significance level was set to *p* > 0.05 after threshold-free cluster estimation with 5000 iterations (non-parametric permutation test).

a The opposite contrast of BPD > HC showed no suprathresholded cluster.

Bidirectional group comparisons between HC and BPD were also conducted for parameter estimate maps of single regressors (go, oddball, no-go) separately, revealing only significant differences for the HC > BPD contrast (see Table 2b). There were no differences in brain activation between the groups when processing go trials. During oddball trials, HC revealed higher left-hemispheric activation in two clusters: a prefrontal cluster comprising the IFG pars orbicularis, middle frontal and precentral gyri (voxels = 68; *t* = 3.99; *p* < 0.04; peak: *x* = −40, *y* = 8, *z* = 30). The second posterior cluster covered broad brain areas of supramarginal, postcentral, superior parietal, angular gyri, the precuneus, and the occipital gyrus (voxels = 2696; *t* = 5.01; *p* = 0.01; peak: *x* = −30, *y* = −40, *z* = 36). During no-go trials, HC showed significantly higher brain activation compared to BPD in three left-hemispheric clusters: a parietal cluster comprising precuneus, superior parietal lobe, and the postcentral gyrus (voxels = 25; *t* = 4.01; *p* < 0.05; peak: *x* = −16, *y* = −52, *z* = 54); a second parietal cluster comprising the supramarginal and superior parietal gyri, and the postcentral gyrus (voxels = 119; *t* = 4.92; *p* < 0.03; peak: *x* = −28, *y* = −40, *z* = 36); and a cluster in the precentral and postcentral gyri (voxels = 141; *t* = 4.20; *p* < 0.04; peak: *x* = −52, *y* = −6, *z* = 34).

### Co-morbidity analyses and correlations between brain activation and self-reports

We were interested in potential confounding effects of comorbid major depression (*n* = 18), a comorbid history for substance use disorder (*n* = 17), and medication effects (*n* = 26). Therefore, we compared patients with BPD having these comorbidities with patients who do not have them using non-parametric statistics of independent two-sample *t* tests. None of the group-comparisons revealed significant differences between these subgroups of BPD patients.

Correlations between BPD self-reported symptom severity and percent signal change at the peak voxels of activity identified by the second-level between-group contrast comparisons (i.e. BPD *v.* HC for no-go > go and oddball > go; Fig. 2) are presented in online Supplementary Table S1. Within the BPD patient group, symptoms measured with the BSL-23 correlated positively with the right pIFG/MFG during no-go > go (*r* = 0.31 *p_FDR_* < 0.03), and the BIS-11 total score (*r* = −0.25 *p_FDR_* < 0.05) and the attentional impulsiveness subscale (*r* = −0.32 *p_FDR_* < 0.03) correlated negatively with the left IFG/MFG during the oddball > go contrast. Correlations within the HC group and both groups collapsed are presented in online Supplementary Table S1. As expected, BPD exhibited significantly higher impulsiveness than HC (*t* = 4.89, *p* < 0.01; see Table 1), and notably, this was inversely correlated with prefrontal brain activation within the patient group.

## Discussion

Consistent with our expectations, patients with BPD showed lower brain activation for the combined response inhibition and attentional saliency contrast (no-go > go) in a cluster comprising the right IFG and posterior parts of the right vlPFC, but not the SMA or pre-SMA. Covering Brodmann areas 44/45, the significant cluster overlaps mostly with the pIFG, which is more directly implicated in response inhibition (Chikazoe, 2010). Corroborating our hypothesis regarding the attentional saliency alone contrast (oddball > go), significantly lower brain activation was found in BPD compared to HC in three left-hemispheric clusters comprising the frontal pole, IFJ, MFG, frontal operculum, but not for medial and mesial brain areas or ACC. However, patients with BPD also showed significantly lower brain activation in midline structures, including the precuneus and mesial parts of the superior parietal lobule. The direct comparison of response inhibition and attentional saliency – for which we had no *a priori* hypotheses – did not reveal any between-group differences, while the mean within group activations covered brain areas comparable to other studies (Chikazoe et al., 2009; Rubia et al., 2003; Schmitz et al., 2006). Furthermore, higher BPD symptoms were correlated with greater right pIFG/MFG activity for the response inhibition and attentional saliency contrast (no-go > go), whereas attentional impulsiveness was inversely correlated with activity in the left IFJ/MFG associated with attentional saliency alone (oddball > go).

Most notably, BPD patients in our sample revealed lower brain activation in two sub-regions of the vlPFC bilaterally, when response inhibition and attentional saliency was analyzed separately. Even though we did not see differences between HC and BPD patients in the direct comparison of response inhibition and attentional saliency (no-go > oddball), BPD patients engaged the right pIFG less during no-go > go and the left IFJ during oddball > go. In applying a similarly modified GNG task, within the right vlPFC, Chikazoe et al. (2009) functionally related the pIFG to saliency processing of behaviorally relevant stimuli during response inhibition and the IFJ to saliency processing independent of the behavioral relevance of the stimuli. As the right pIFG has been described in both response inhibition and attentional saliency (Walther et al., 2011) and, according to Corbetta and Shulman (2002), it has a crucial role with links to both right-lateralized dorsal and ventral attentional networks, we interpret the lower engagement in BPD during no-go > go as a neural correlate of attentional dysfunctions during response inhibition. Saliency processing is one aspect among others of cognitive control, which could underlie difficulties with response inhibition, and these frontoparietal networks are also altered in many forms of psychopathology, including ADHD, major depression, and schizophrenia (Marek & Dosenbach, 2018).

The IFJ reflects a general node in reorienting attention, which is active during no-go and similarly infrequent go trials (Chikazoe, 2010). We found lower brain activation in the left IFJ in patients with BPD when processing saliency alone (oddball > go), which is comparable with the ‘infrequent go > frequent go’ contrast reported by Chikazoe (2010). In addition to that lower left vlPFC brain activation when isolating attentional saliency in the oddball > go contrast, BPD patients also revealed lower neural engagement in several other frontoparietal regions of the left hemisphere. We had no specific hypothesis regarding activation differences between BPD and HC for the oddball > go contrast, but in addition to the predominantly right-sided evidence of vlPFC involvement during response inhibition, human lesion studies have found that also the left vlPFC is critical for successful response inhibition (Swick, Ashley, & Turken, 2008). The exhibited lower left-lateralized attention-related engagement in BPD we see could be a sign of an interhemispheric imbalance. Disruptions of interhemispheric connectivity in frontoparietal networks have been correlated with behavioral deficits in other clinical groups (He et al., 2007).

BPD patients in our study revealed similar behavioral performances, which is in line with previous studies (van Eijk et al., 2015). However, albeit comparable accuracies in task performance, there is behavioral evidence showing faster reaction times (Hagenhoff et al., 2013; Rentrop et al., 2008), and also studies showing performance differences when applying emotional neutral GNG tasks in BPD (Cackowski et al., 2014; Coffey, Schumacher, Baschnagel, Hawk, & Holloman, 2011; Mortensen et al., 2010). Altered motor impulsivity has also been proposed as an endophenotype in BPD (McCloskey et al., 2009). van Eijk et al. (2015) found no performance nor reaction time differences compared to HC in two samples of BPD patients. The used GNG task in their sample one had 29% no-go trials without an oddball condition. In sample two they used a hybrid response inhibition task with 12.5% no-go trials and an interference condition. Our BPD group showed a trend toward poorer performances, which was negatively correlated with more BPD symptom load, and especially more self-rated attentional impulsiveness. This is in contrast to other studies showing no correlation between self-ratings and behavioral measures of impulsivity (Jacob et al., 2010; Stahl et al., 2014). Moreover, self-ratings of BPD symptoms correlated with attentional saliency and response inhibition-related brain activation in the right vlPFC, and self-rated attentional impulsiveness correlated negatively with the left vlPFC for the attentional saliency contrast. These brain-behavioral correlations add evidence to the notion that patients with BPD have disrupted attentional processing during response inhibition, which may underlie the clinically limited impulse control and attentional capacities (Cole, Repovs, & Anticevic, 2014; Davids & Gastpar, 2005).

### Limitations

Several limitations of the study should be considered. The patients with BPD were hospitalized and highly comorbid, with 58% being under stable medication and 37.8% having a history of substance use. This can be considered a naturalistic inpatient population in a typical psychiatric clinic for BPD in Switzerland. Nevertheless, multiple procedures were in place to minimize the impacts of substance use on the neuroimaging results: alcohol/substance use (except nicotine) was not permitted beginning 3 days before the scan day, and participants were required to have a negative urine toxicology screen and a breathalyzer test to be scanned. The impact of subthreshold ADHD symptoms could not be ascertained, as no dimensional measure of ADHD symptom was included in the study. However, all patients underwent a SCID-I interview, the clinician attended regular consensus diagnostic meetings with a senior psychiatrist who also regularly saw the patients, and based on these assessments, no patient received an ADHD diagnosis (see Table 1). Hyperconnectivity between basal ganglia and salience regions in ADHD has recently been shown (Damiani et al., 2020). Nevertheless, we did not find an overlap between significant brain regions of our saliency contrast and the regions described by Damiani et al. (2020). The event-related analysis may have been underpowered with only 23% of all trials (oddball and no-go), which should be further explored in future research.

It is important to consider that attentional saliency and response inhibition could potentially support social-cognitive functions relevant to personality disorder (PD), such as self-referential processes (Scalabrini, Mucci, & Northoff, 2018). Indeed, impairments in self and interpersonal functioning are central to proposed alternative dimensional PD diagnoses, such as in the DSM-5 Alternative Model for PDs (Widiger, 2011) and the International Classification of Diseases – 11th Revision (Tyrer et al., 2011). Accordingly, future research should be directed toward understanding how personality disturbances in self and interpersonal functioning are represented in the brain, including in brain systems involved in self-referential processes, such as the default-mode network (Aguilar-Ortiz et al., 2020). Similarly, the results of the present investigation speak to the potential utility of incorporating dimensional assessments of pathological personality trait domains into neurobiological research on PD, as we determined that an impulsiveness dimension was significantly associated with activation in prefrontal brain regions. Relationships between pathological personality traits and potential neuroimaging-based biomarkers should be explored in future research, as this work could illuminate the neurobiology of traits contained in proposed dimensional conceptualizations of PD according to the National Institute of Mental Health's Research Domain Criteria initiative (Koudys et al., 2019).

## Conclusion

To our knowledge, we are the first to uncover differential patterns of neural activation associated with response inhibition and attentional saliency in patients with BPD compared to HCs. Using a modified GNG task with infrequent oddball trials, we sought to disentangle partially overlapping neural processes that may have been obscured in previous studies of response inhibition in BPD using conventional GNG tasks. Compared to HC, lower brain activation in BPD patients was found during attentional saliency and response inhibition in the right vlPFC and when isolating saliency processing, in three left-lateralized clusters of prefrontal, lateral parietal, and posterior midline structures. During response inhibition, BPD patients may have trouble in filtering salient but behaviorally irrelevant stimuli from relevant ones via the right vlPFC. We conclude that disrupted response inhibition processes in BPD are related in part to the inefficient engagement of frontoparietal brain regions involved in attentional saliency.

## Supplementary material

For supplementary material accompanying this paper visit https://doi.org/10.1017/S0033291721000118. click here to view supplementary material
