# Supplementary material for: Attentional salience and the neural substrates of response inhibition in borderline personality disorder
Source: Psychol Med. 2021 Mar 16;52(15):3451–9. doi: 10.1017/S0033291721000118 (PMC9772916; doi:10.1017/S0033291721000118)
Supplement: Supplementary file 1 [file S0033291721000118sup001.docx]

**Supplementary material**

Table S1

1. *Correlations between Symptomatology and significant Brain Clusters groups collapsed (N=74)*

|  | No-Go>Go | Odd>Go |  |  |
| --- | --- | --- | --- | --- |
|  | Right IFG/MFG | Left FP/MFG | Left IFG/MFG | Left SPL |
| BSL-23 | *r*=-.19 *p<*.11 | *r*=-.22 *p*=.07 | *r*=-.35 *p*<.01**  ***p_FDR_<.01***** | *r*=-.31 *p*<.01**  ***p_FDR_<.02**** |
| BIS-11: sum | *r*=-.28 *p*<.02*  ***p_FDR_=.03**** | *r*=-.20 *p*=.09 | *r*=-.34 *p<.01** p****_FDR_<.02**** | *r*=-.23 *p=.05* |
| attention | *r*=-.24 *p*<.05*  ***p_FDR_<.05**** | *r*=-.36 *p*<.01**  ***p_FDR_<.01***** | *r*=-.38 *p<.01***  ***p_FDR_<.01***** | *r*=-.27 *p=.02**  ***p_FDR_=.03**** |
| motor | *r*=-.13 *p*=.27 | *r*=-.03 *p*=.86 | *r*=-.18 *p=.13* | *r*=-.16 *p=.18* |
| non-planning | *r*=-.32 *p*<.01**  ***p_FDR_=.01**** | *r*=-.14 *p=.24* | *r*=-.31 *p<.01***  ***p_FDR_<.02**** | *r*=-.17 *p<.15* |

1. *Correlations between Symptomatology and significant Brain Clusters in HC (N=29)*

|  | No-Go>Go | Odd>Go |  |  |
| --- | --- | --- | --- | --- |
|  | Right IFG/MFG | Left FP | Left IFG/MFG | Left SPL |
| BSL-23 | *r*=.15 *p=*.23 | *r*=.11 *p*=.29 | *r*=.40 *p*<.02*  ***p_FDR_<.03**** | *r*=.30 *p*<.06 |
| BIS-11: sum | *R*<-.01 *p*<.50 | *R*<.03 *p*<.45 | *r*=-.04 *p=.42* | *r*=.24 *p=.11* |
| attention | *r*=.10 *p*=.30 | *r*=-.13 *p*=.26 | *r*=.12 *p=.26* | *r*=.33 *p<.05**  ***p_FDR_<.05**** |
| motor | *r*=.11 *p*=.29 | *r*=.14 *p*=.25 | *r*=-.05 *p=.41* | *r*=-.02 *p=.46* |
| non-planning | *r*=-.13 *p*=.26 | *r*<.05 *p=.41* | *r*=-.12 *p=.27* | *r*=.27 *p=.08* |

1. *Correlations between Symptomatology and significant Brain Clusters in BPD (N=45)*

|  | No-Go>Go | Odd>Go |  |  |
| --- | --- | --- | --- | --- |
|  | Right IFG/MFG | Left FP | Left IFG/MFG | Left SPL |
| BSL-23 | *r*=.31 *p=*.02*  ***p_FDR_=.03**** | *r*=.01 *p*=.26 | *r*=-.18 *p*<.20 | *r*=.16 *p*=.46 |
| BIS-11  sum | *r*=-.11 *p*=.24 | *r*=-.05 *p*<.38 | *r*=-.25 *p<.05*  ***p_FDR_<.05**** | *r*=-.15 *p<.17* |
| attention | *r*=.07 *p*=.32 | *r*=-.20 *p=*.09 | *r*=-.32 *p<.02**  ***p_FDR_=.03**** | *r*=-.13 *p=.19* |
| motor | *r*=-.09 *p*=.28 | *r*=.04 *p*=.40 | *r*=-.12 *p=.21* | *r*=-.09 *p=.28* |
| non-planning | *r*=-.20 *p*<.01 | *r*=-.01 *p=.48* | *r*=-.22 *p=.08* | *r*=-.15 *p=.17* |

*Note*. Correlations between individual first-level contrast percent-signal changes of the significant second-level between group clusters and individual self-ratings. False discovery rate (FDR) for multiple testing was calculated according to Benjamin and Hochberg (1995). BIS-11 = Barratt Impulsiveness Scale (Patton et al., 1995); BPD = Borderline personality disorder; BSL-23 = Borderline Symptom List-23 (Bohus et al., 2009); FP = frontal pole; HC = healthy controls; IFG = inferior frontal gyrus; MFG = middle frontal gyrus; SPL = superior parietal lobule.

| Table S2. |  |  |  |  |
| --- | --- | --- | --- | --- |
| *Regression of performance on brain activation.* | | | |  |
| right pIFG/MFG (nogo > go contrast) | | | |  |
|  |  | Beta | adjusted R^2^ | *p* |
| model |  |  | .07 | *.076* |
|  | go performance | .497 |  | .006* |
|  | oddball performance | .100 |  | .583 |
|  | nogo perfomrance | -.132 |  | .322 |
|  | go reaction time | .573 |  | .020* |
|  | oddball reaction time | -.129 |  | .465 |
|  |  |  |  |  |
| left FP/MFG (oddball > go contrast) | | |  |  |
|  |  | Beta | adjusted R^2^ | *p* |
| model |  |  | .048 | .139 |
|  | go performance | .082 |  | .645 |
|  | oddball performance | .168 |  | .366 |
|  | nogo perfomrance | .211 |  | .119 |
|  | go reaction time | .123 |  | .614 |
|  | oddball reaction time | .086 |  | .631 |
|  |  |  |  |  |
| left IFG/MFG/PrCG (oddball > go contrast) | | | |  |
|  |  | Beta | adjusted R^2^ | *p* |
| model |  |  | .006 | .377 |
|  | go performance | .114 |  | .431 |
|  | oddball performance | .213 |  | .263 |
|  | nogo perfomrance | .086 |  | .533 |
|  | go reaction time | .342 |  | .173 |
|  | oddball reaction time | -.043 |  | .814 |
|  |  |  |  |  |
| left SPL/SMG (oddball > go contrast) | | | |  |
|  |  | Beta | adjusted R^2^ | *p* |
| model |  |  | .019 | .279 |
|  | go performance | .379 |  | .040* |
|  | oddball performance | .045 |  | .810 |
|  | nogo perfomrance | -.002 |  | .986 |
|  | go reaction time | .271 |  | .276 |
|  | oddball reaction time | -.050 |  | .781 |
| *Note*. There was no reaction time for nogo trials as participants had to withhold their reaction; pIFG/MFG=posterior inferior-frontal and middle-frontal gyri; FP/MFG=frontal pole and middle-frontal gyrus; IFG/MFG/PrCG=inferior-frontal, middle-frontal, and precentral gyri; *=significant result. | | | | |
